# Status of Immunotherapy Acceptance in Chinese Patients With Multiple Sclerosis: Analysis of *Multiple Sclerosis Patient Survival Report 2018*

**DOI:** 10.3389/fneur.2021.651511

**Published:** 2021-04-08

**Authors:** Ran Zhou, Qiuming Zeng, Huan Yang, Yan Xu, Guojun Tan, Hongbo Liu, Lihua Wang, Hongyu Zhou, Meini Zhang, Jinzhou Feng, Tao Jin, Xinghu Zhang, Jiawei Wang, Xu Zhang, Feng Gao, Chunsheng Yang, Bitao Bu, Chunyang Li, Min Zhang, Huiqing Dong, Aiyu Lin, Weibin Liu, Lei Wu, Manxia Wang, Yulan Tang, Honghao Wang, Youming Long, Zhe Wang, Weihong Zheng

**Affiliations:** ^1^Department of Neurology, Xiangya Hospital, Central South University, Changsha, China; ^2^Department of Neurology, Peking Union Medical College Hospital, Chinese Academy of Medical Sciences and Peking Union Medical College, Beijing, China; ^3^Department of Neurology, The Second Hospital of Hebei Medical University, Shijiazhuang, China; ^4^Department of Neurology, The First Affiliated Hospital of Zhengzhou University, Zhengzhou, China; ^5^Department of Neurology, The Second Affiliated Hospital, Harbin Medical University, Harbin, China; ^6^Department of Neurology, West China Hospital, Sichuan University, Chengdu, China; ^7^Department of Neurology, The First Affiliated Hospital of Shanxi Medical University, Taiyuan, China; ^8^Department of Neurology, The First Affiliated Hospital of Chongqing Medical University, Chongqing, China; ^9^Department of Neurology and Neuroscience Center, The First Hospital of Jilin University, Changchun, China; ^10^Department of Neurology, Beijing Tiantan Hospital, Capital Medical University, Beijing, China; ^11^Department of Neurology, Beijing Tongren Hospital, Capital Medical University, Beijing, China; ^12^Department of Neurology, The First Affiliated Hospital of Wenzhou Medical University, Wenzhou, China; ^13^Department of Neurology, Peking University First Hospital, Beijing, China; ^14^Department of Neurology, Tianjin Medical University General Hospital, Tianjin, China; ^15^Department of Neurology, Tongji Hospital, Tongji Medical College, Huazhong University of Science and Technology, Wuhan, China; ^16^Department of Neurology, The Affiliated Hospital of Inner Mongolia Medical University, Hohhot, China; ^17^Department of Neurology, Xuanwu Hospital, Capital Medical University, Beijing, China; ^18^Department of Neurology, The First Affiliated Hospital of Fujian Medical University, Fuzhou, China; ^19^Department of Neurology, The First Affiliated Hospital of Sun Yat-sen University, Guangzhou, China; ^20^Department of Neurology, General Hospital of the People's Liberation Army, Beijing, China; ^21^Department of Neurology, The Second Hospital of Lanzhou University, Lanzhou, China; ^22^Department of Neurology, The First Affiliated Hospital, Guangxi Medical University, Nanning, China; ^23^Department of Neurology, Nanfang Hospital, Southern Medical University, Guangzhou, China; ^24^Department of Neurology, The Second Affiliated Hospital of Guangzhou Medical University, Guangzhou, China; ^25^Department of Neurology, The First Affiliated Hospital, Dalian Medical University, Dalian, China; ^26^Department of Neurology, Affiliated Zhongshan Hospital, Xiamen University, Xiamen, China

**Keywords:** multiple sclerosis, multicenter study, disease-modifying therapies, immunosuppressive drug, drug selection

## Abstract

**Objective:** The prevalence of multiple sclerosis (MS) in China is low, although it has been increasing recently. Owing to the paucity of data on immunotherapy acceptance in the Chinese population, we conducted this study to analyze factors affecting the acceptance of immunotherapy and selection of disease-modifying therapies (DMTs) based on personal and clinical data of patients with MS.

**Methods:** In this study, data were obtained from the *Multiple Sclerosis Patient Survival Report 2018*, which was the first national survey of patients with MS in China. There were 1,212 patients with MS from 31 provinces who were treated at 49 Chinese hospitals over a 4-month period from May 2018 to August 2018, and the patients were asked to complete online questionnaires to assess their understanding of the disease.

**Results:** In general, highly educated patients with frequent relapses were more willing to receive treatment regardless of DMTs or other immunotherapy, and patients with more understanding of the disease opted to be treated. Younger patient population, patients with severe disease course, and those with more symptoms were likely to choose the treatment. Moreover, a higher proportion of women chose to be treated with DMTs than with other immunotherapies.

**Conclusions:** Education status and patient awareness of the disease impact the treatment acceptance in Chinese patients with MS. Therefore, we call for improving the awareness of MS disease and social security to help patients to improve their quality of life.

## Introduction

Idiopathic inflammatory demyelinating diseases (IIDDs) constitute a group of disorders, such as multiple sclerosis (MS) and neuromyelitis optica spectrum disorder (NMOSD), characterized by inflammatory lesions that are associated with loss of myelin and eventually axonal damage. MS, as the most studied subgroup of IIDDs, affects more than 2.8 million people worldwide (1). It is a leading cause of disability among young adults and has a poor prognosis, with about half the patients requiring permanent use of a wheelchair at 25 years after diagnosis (2). MS has four distinct subtypes, namely, clinically isolated syndrome (CIS), relapsing remitting MS (RRMS), secondary progressive MS (SPMS), and primary progressive MS (PPMS) (3). To date, since the introduction of the first immunomodulating interferon-β-1b in 1993, the United States Food and Drug Administration (US FDA) has approved a total of 15 disease-modifying therapies (DMTs) for the treatment of MS (4). The DMTs include subcutaneous injection medications [interferon-β-1b, interferon-β-1a (two formulations), peg-interferon-β-1a, and glatiramer acetate], intravenous injection medications (alemtuzumab, natalizumab, ocrelizumab, and mitoxantrone), and oral medications [ozanimod, dimethyl fumarate (two formulations), cladribine, siponimod, fingolimod, and teriflunomide]. In the past 20 years, disease-modifying therapy (DMT) has been proposed to modify the disease course, reduce relapses, slow the course of disability progression, manage ongoing symptoms, and restore the quality of life with a favorable safety and efficacy profile for patients with MS (5).

NMOSD was long believed to be an aggressive form of MS, but with the in-depth understanding of the pathogenesis, serum and cerebrospinal fluid detection, especially the detection of aquaporin-4 antibody, and magnetic resonance imaging features have been widely used to distinguish the two diseases. In China, epidemiological data indicated that the ratio of annual incident cases of MS to NMOSD was 1:1.2 (6), and the incidence of NMOSD per 100,000 person years was 0.278, with 0.075 in children and 0.347 in adults (7). Therefore, patients with NMOSD were not included in our study. Furthermore, previous studies have shown women to be more affected by MS than men, with the female-to-male ratio varying between 1.5:1 and 2.5:1 (8). The distribution pattern of MS is heterogeneous, with its highest prevalence in North America (140/100,000 population) and Europe (108/100,000 population) and the lowest in East Asia (2.2/100,000 population) and sub-Saharan Africa (2.1/100,000 population) (9). This uneven distribution of MS across populations can be attributed to differences in genes and the environment and their interaction (10). Asians are known to have a lower incidence of MS, which was observed in Chinese adults to be 0.288 per 100,000 population between 2016 and 2018 (6). However, although the prevalence of MS in China is low, the number of patients with MS cannot be underestimated because of the large population base in China.

Before 2015, IFN-β, oral corticosteroids, and other immunotherapy treatments were the initial choice of drugs for Chinese patients with MS. The advent of Betaferon (trade name of recombinant human interferon-β-1b injection) in the Chinese market brought the concept of DMTs. However, shortage of drug supply due to various reasons causes helplessness and sense of panic in patients with MS. Therefore, given that no disease-modifying drugs were available in China, neurologists had no choice but to prescribe other immunosuppressive drugs to control disease progression as the literature supports the role of other immunotherapy drugs in the treatment of MS, such as azathioprine (11), mycophenolate mofetil (12), and rituximab (13). The prices of oral corticosteroids and most immunosuppressive drugs, including azathioprine and mycophenolate mofetil, are affordable for most patients, and the guarantee of national health insurance for these patients is also strong enough for patients to maintain treatment adherence. The *Multiple Sclerosis Patient Survival Report 2018* was the first nationwide survey on the immunotherapy acceptance status of Chinese patients with MS, which revealed the status of patients with MS in China from multiple dimensions for the first time. In the present study, we aimed to investigate the acceptance of disease treatment by patients with MS and analyze factors affecting treatment acceptance in China so as to provide recommendations for clinicians to treat patients with MS and help patients improve their quality of life.

## Materials and Methods

### Study Design and Patients

The *Multiple Sclerosis Patient Survival Report 2018* was a national and multicenter study in patients with MS, and the survey covered five aspects: basic characteristics of patients, disease characteristics, diagnosis and treatment, psychological burden, and economic burden. This was the first survey to reveal the survival status of patients with MS in China from multiple dimensions. The study population consisted of 1,362 patients with MS from 31 provinces who were treated at 49 Chinese comprehensive hospitals over a period of 4 months from May 2018 to August 2018. The diagnosis of the included patients was based on the 2017 Revisions of the McDonald criteria (14). The data for the present study were obtained from the *Multiple Sclerosis Patient Survival Report 2018*, and given that the purpose of our study is to explore the patient's acceptance of the treatment, we excluded patients with missing detailed treatment information. Hence, the final analysis population consisted of 1,212 patients with MS. The study was approved by the Medical Research Ethics Committee of Xiangya Hospital and conducted in accordance to the Declaration of Helsinki.

### Research Indicators

The present study used medical records of patients with MS for retrospective examination. Analysis was based on patient characteristics, clinical information, and other characteristics. Patient characteristics included patient's age, sex, marital status, fertility status, and education level. Clinical information included course of disease, diagnosis, clinical symptoms, and the number of symptoms. Other characteristics included emotional status, employment status, and assessment of disease awareness through 10 questions about MS.

### Statistical Analysis

All the statistical data were analyzed by SPSS 26.0 software. First, patients with MS were divided into two groups depending on whether they received therapy or not, and then univariate and multivariate logistic regression analyses were performed for patient characteristics, clinical information, and other characteristics between these two groups. Second, the treated patients were divided into two groups depending on whether they received DMTs or not, and univariate logistic regression analysis was performed on patient characteristics, clinical information, and other characteristics in these two groups. In addition, to analyze the characteristics of patients using DMTs, we further compared patients who received DMTs with untreated patients. The three quantitative data, namely, age, Expanded Disability Status Scale (EDSS), and course of disease, were expressed as median and interquartile range (IQR), and independent-sample non-parametric tests were used for comparison between the groups. Sex, marital status, fertility status, education status, disease subtype, and clinical symptoms are expressed as rates, and the chi-square test was used to determine between-group differences. In addition, the odds ratio (OR) was used as a measure of association between exposure and an outcome. Two events are considered independent if and only if the OR is equal to 1, and these two events are considered correlated with each other if the OR is >1. Conversely, if the OR is <1, then the two events are negatively correlated, and the presence of one event reduces the odds of the other event. The 95% confidence interval (CI) is a type of estimate computed from the statistics of the observed data, and the interval has an associated confidence level that the true parameter is in the proposed range. All statistics were two-sided, and *P* < 0.05 was considered statistically significant, whereas *P* < 0.1 was considered as the criteria for multivariate logistic regression analysis.

## Results

### Reasons for Not Receiving Treatment

**Figure 1:**
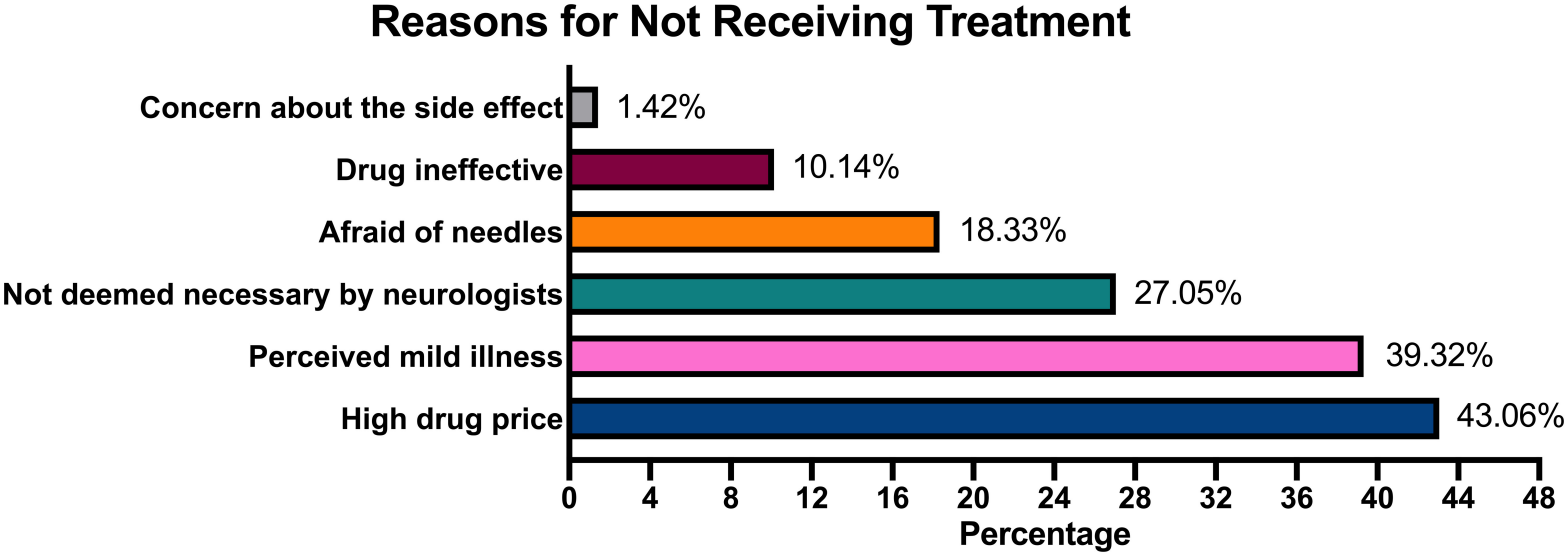
Reasons for not receiving treatment.

A 4-month survey of 49 hospitals interviewed ~1,212 patients with MS, of whom 324 patients with MS accepted treatment and merely 111 patients with MS accepted the treatment with DMTs, which indicates that ~26.73% of patients with MS accepted treatment and 9.1% of patients with MS were treated with DMTs after diagnosis. When the reasons for not receiving treatment were analyzed, patients cited multiple choices based on personal situation. About 43.06% of the patients blamed the refusal of treatment on economic burden, whereas 39.32% considered their symptoms mild and hence refused the treatment. Moreover, 27.05% of the patients did not receive treatment because their admitting physician believed that the treatment was not required. Refusal of treatment for drug-related reasons included long-term drug injection (18.33%), drug ineffectiveness (10.14%), and side effects (<2%; Figure 1).

### Treated Group vs. Untreated Group

#### Patient Characteristics

We divided 1,212 patients into two groups (treated group and untreated group) according to whether they received immunotherapy or not (Table 1). The treated group consisted of patients who have received DMTs or other immunotherapy, and the untreated group included patients who did not receive any treatment or have only been treated with Chinese medicine. There were 216 women and 102 men in the treated group and 575 women and 285 men in the untreated group. There was no significant relationship between sex composition, procreation situation (fertility or not), and whether the patients received treatment. However, age, marital status, and educational level were associated with drug acceptance. Unmarried patients were 1.447 (95% CI: 1.065–1.966) times more likely to be treated than married patients. Considering the education level, patients with a bachelor's degree were 1.457 (95% CI: 0.871–2.437) times more willing to choose medication than patients with primary education.

**Table 1:** Patient Characteristics in the treated group and untreated group.

**Variables**	**Untreated group (*N* = 888)**	**Treated group (*N* = 324)**	**Odds ratio (95% confidence interval)**	***P*-value**
Sex (female%)	66.87% (575/860)	67.92% (216/318)	0.953 (0.724–1.254)	*P* > 0.05
Age, median (IQR), years	41.42 (18.58) (*N* = 798)	38.97 (18.79) (*N* = 310)	0.989 (0.979–0.999)	*P* = 0.027
Married (%)	80.57% (647/803)	74.13% (235/317)	1.447 (1.065–1.966)	*P* = 0.018
Procreation (%)	74.58% (584/783)	69.87% (218/312)	1.288 (0.968–1.715)	*P* > 0.05
Education level (%)				*P* < 0.0001
Primary education	7.94% (61/768)	7.72% (24/311)	1	
Junior high school	29.43% (226/768)	19.61% (61/311)	0.686 (0.396–1.190)	
High school	29.69% (228/768)	26.05% (81/311)	0.903 (0.528–1.543)	
Bachelor's degree	32.94% (253/768)	46.62% (145/311)	1.457 (0.871–2.437)	

*IQR, interquartile range*.

#### Disease Characteristics

Except for the course of the disease and EDSS scores, the difference in the diagnosis and number of symptoms was associated with drug acceptance (Table 2). Patients diagnosed with RRMS, SPMS, and PPMS were 2.279 (95% CI: 1.406–3.696), 3.318 (95% CI: 1.454–7.574), and 1.896 (95% CI: 0.572–6.284) times, respectively, more willing to accept medication than patients with CIS. A total of 14 symptoms, namely, hypoxia, amblyopia, limb weakness, dizziness, emotion disorder, unstable walking, muscle spasms, pain, sexual dysfunction, dysphonia, fecal and urine incontinence, paresthesia, memory impairment, and fatigue were considered in the study, all of which had a much higher probability of appearing in the treated group than in the untreated group, indicating that patients with more disease symptoms would be more prone to accept medication.

**Table 2:** Disease characteristics in the treated group and untreated group.

**Variables**	**Untreated group (*N* = 888)**	**Treated group (*N* = 324)**	**Odds ratio (95% confidence interval)**	***P*-value**
Course of diseases, median (IQR), years	6.15 (6.59) (*N* = 569)	6.73 (6.71) (*N* = 259)	1.023 (0.997–1.048)	*P* > 0.05
**Diagnosis (%)**				*P* = 0.005
CIS	22.67% (146/644)	11.22% (22/196)	1	
RRMS	71.43% (460/644)	80.61% (158/196)	2.279 (1.406–3.696)	
SPMS	3.73% (24/644)	6.12% (12/196)	3.318 (1.454–7.574)	
PPMS	2.17% (14/644)	2.05% (4/196)	1.896 (0.572–6.284)	
EDSS, median (IQR)	2 (2.5) (*N* = 566)	2 (4) (*N* = 161)	1.001 (0.999–1.004)	*P* > 0.05
**Symptoms (%)**				
Hypoxia	25.90% (230/888)	45.68% (148/324)	2.404 (1.845–3.135)	*P* < 0.0001
Amblyopia	17.55% (156/888)	29.32% (95/324)	1.961 (1.460–2.639)	*P* < 0.0001
Limb weakness	40.09% (356/888)	64.51% (209/324)	2.717 (2.083–3.534)	*P* < 0.0001
Dizziness	23.76% (211/888)	33.02% (107/324)	1.582 (1.198–2.092)	*P* = 0.001
Emotional disorder	17.57% (156/888)	37.35% (121/324)	2.793 (2.105–3.717)	*P* < 0.0001
Unstable walking	27.70% (246/888)	51.85% (168/324)	2.809 (2.160–3.650)	*P* < 0.0001
Muscle spasms	11.15% (99/888)	30.56% (99/324)	3.509 (2.558–4.808)	*P* < 0.0001
Pain	16.10% (143/888)	21.30% (69/324)	1.410 (1.024–1.942)	*P* = 0.036
Sexual dysfunction	4.84% (43/888)	10.19% (33/324)	2.227 (1.389–3.571)	*P* = 0.001
Dysphonia	9.80% (87/888)	19.14% (62/324)	2.178 (1.529–3.106)	*P* < 0.0001
Fecal and urine incontinence	16.67% (148/888)	32.41% (105/324)	2.398 (1.789–3.205)	*P* < 0.0001
Paresthesia	42.91% (381/888)	60.19% (195/324)	2.012 (1.553–2.604)	*P* < 0.0001
Memory impairment	14.64% (130/888)	28.40% (92/324)	2.315 (1.704–3.135)	*P* < 0.0001
Fatigue	30.07% (267/888)	52.16% (169/324)	2.538 (1.953–3.289)	*P* < 0.0001
**Number of symptoms**			1.245 (1.193–1.298)	*P* < 0.0001
<2	36.49% (324/888)	10.49% (34/324)		
≥2 to <4	31.53% (280/888)	26.23% (85/324)		
≥4 to <8	23.09% (205/888)	40.12% (130/324)		
≥8	8.89% (79/888)	23.16% (75/324)		

*IQR, interquartile range; CIS, clinically isolated syndrome; RRMS, relapsing remitting multiple sclerosis; SPMS, secondary progressive multiple sclerosis; PPMS, primary progressive multiple sclerosis; EDSS, Expanded Disability Status Scale*.

#### Other Characteristics

**Figure 2:**
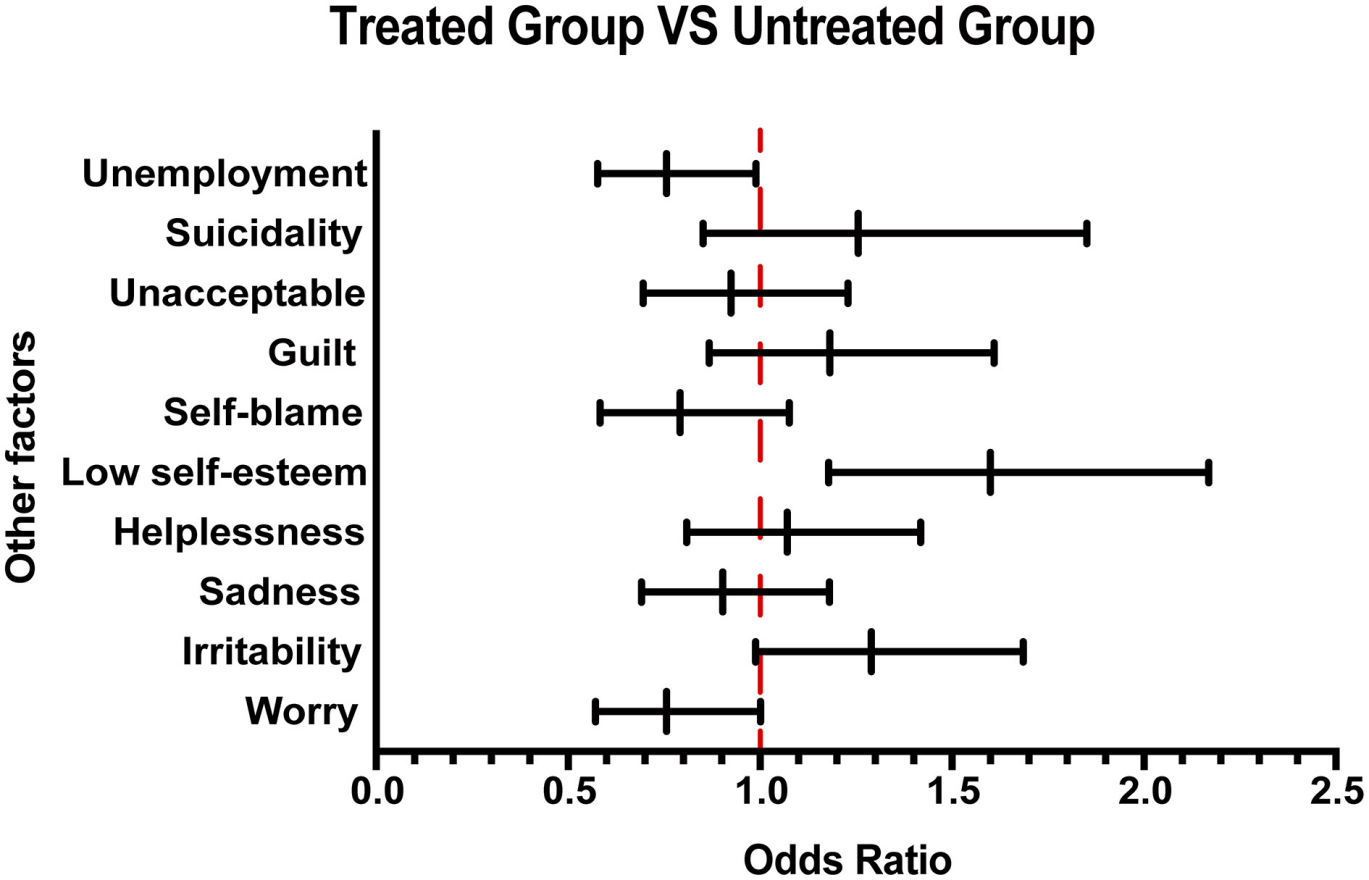
Other emotional and employment factors differing between the treated and the untreated groups. The forest plot shows odds ratio differences in other factors between the treated and untreated groups.

In this survey, in addition to exploring the basic and disease characteristics of Chinese patients with MS, we conducted a 10-question survey to assess the patient's understanding of the disease. From the survey, we found that patients in the group that received treatment generally scored higher than those in the group that did not receive treatment (Supplementary Table 1). MS is a chronic neurological disease with dramatic impact on a patient's mental well-being (15). In our survey, 83.17% (1,008/1,212) of patients with MS were experiencing emotional problems. The psychological and economic burden of the disease will directly influence the acceptance of medication. Therefore, in this research, the assessment of psychological burden was conducted considering several emotional states such as worry, irritability, sadness, helplessness, low self-esteem, self-blame, guilt, unacceptance, and suicidality. After analyzing the data, as shown in Figure 2, a greater proportion of low self-esteem was observed in the treated group than in the untreated group. Moreover, in terms of employment status, we found that the unemployment rate of patients not receiving treatment was higher than that of patients receiving treatment, suggesting that the patients' income affects the drug acceptance.

#### Multivariate Analysis

After the univariate analysis, a multivariate logistic regression analysis was performed for basic characteristics, clinical characteristics, and other factors between the treated and untreated groups, and significant differences were obtained in indicators including education level, disease classification, clinical symptoms such as mood disorders and muscle spasms, emotional response, and some questions to assess the patient's understanding of the disease (Table 3). In the analysis of educational level OR, a patient with a bachelor's degree was 2.210 (95% CI: 0.921–5.306) times more likely to accept treatments than those with primary education. Moreover, in terms of disease classification, patients diagnosed with RRMS, SPMS, and PPMS were 4.018 (95% CI: 1.590–10.152), 4.934 (95% CI: 1.195–20.365), and 2.187 (95% CI: 0.169–28.388) times, respectively, more likely to accept treatments than those with CIS. Furthermore, patients with limb weakness, emotional disorder, muscle spasms, and paresthesia were 1.938 (95% CI: 1.148–3.268), 2.268 (95% CI: 1.279–4.016), 2.732 (95% CI: 1.475–5.076), and 1.766 (95% CI: 1.048–2.974) times, respectively, more likely to accept treatments than those without these symptoms. As for the emotional status, patients with feelings of sadness and unacceptance were less likely to accept the treatment. In terms of MS-related questions, the ORs of questions 4, 6, 8, and 10 were 1.878 (95% CI: 1.082–3.260), 1.812 (95% CI: 1.036–3.165), 1.984 (95% CI: 0.936–4.202), and 1.783 (95% CI: 1.065–2.985), respectively.

**Table 3:** Multivariate logistic regression model of all indicators between the treated group and untreated group.

**Variables**	**Untreated group (*N* = 888)**	**Treated group (*N* = 324)**	**Odds ratio (95% confidence interval)**	***P*-value**
**Education level (%)**				*P* = 0.021
Primary education	7.94% (61/768)	7.72% (24/311)	1	
Junior high school	29.43% (226/768)	19.61% (61/311)	0.831 (0.323–2.139)	
High school	29.69% (228/768)	26.05% (81/311)	1.166 (0.492–2.762)	
Bachelor degree	32.94% (253/768)	46.62% (145/311)	2.210 (0.921–5.306)	
**Diagnosis (%)**				*P* = 0.026
CIS	22.67% (146/644)	11.22% (22/196)	1	
RRMS	71.43% (460/644)	80.61% (158/196)	4.018 (1.590–10.152)	
SPMS	3.73% (24/644)	6.12% (12/196)	4.934 (1.195–20.365)	
PPMS	2.17% (14/644)	2.05% (4/196)	2.187 (0.169–28.388)	
**Symptoms (%)**				
Limb weakness	40.09% (356/888)	64.51% (209/324)	1.938 (1.148–3.268)	*P* = 0.013
Emotion disorder	17.57% (156/888)	37.35% (121/324)	2.268 (1.279–4.016)	*P* = 0.005
Muscle spasms	11.15% (99/888)	30.56% (99/324)	2.732 (1.475–5.076)	*P* = 0.001
Paresthesia	42.91% (381/888)	60.19% (195/324)	1.766 (1.048–2.974)	*P* = 0.033
**Emotional response (%)**				
Sadness	45.14% (311/689)	42.63% (136/319)	0.517 (0.301–0.887)	*P* = 0.017
Unacceptable	33.38% (230/689)	31.66% (101/319)	0.465 (0.269–0.804)	*P* = 0.006
**Multiple sclerosis-related problems (%)**				
Q4	64.90% (442/681)	67.61% (215/318)	1.878 (1.082–3.260)	*P* = 0.025
Q6	61.59% (404/656)	62.07% (198/319)	1.812 (1.036–3.165)	*P* = 0.037
Q8	83.04% (558/672)	93.46% (300/321)	1.984 (0.936–4.202)	*P* = 0.074
Q10	52.06% (341/655)	67.43% (205/304)	1.783 (1.065–2.985)	*P* = 0.028

*CIS, clinically isolated syndrome; RRMS, relapsing remitting multiple sclerosis; SPMS, secondary progressive multiple sclerosis; PPMS, primary progressive multiple sclerosis*.

### DMT Treatment Group vs. Other Immunotherapy Group

Of 324 patients who received the treatment, 110 received interferon-β-1b and were grouped under the DMT treatment group, whereas 208 patients received low-dose glucocorticoids, azathioprine, mycophenolate mofetil (two formulations), or rituximab and were classified into the other immunotherapy group.

There were 85 women and 25 men in the DMT treatment group and 131 women and 77 men in the other immunotherapy group, indicating that female patients are more willing to accept treatment with DMTs than their male counterparts. Most of the basic characteristics such as age, procreation situation, and marital status were not significantly associated with the type of treatment. However, education level influenced patients' treatment choice, and patients with a bachelor's degree were 2.800 (95% CI: 1.051–7.458) times more likely to accept DMTs than patients with primary school education (Table 4).

**Table 4:** Patient characteristics of the DMT treatment group and other immunotherapy group.

**Variables**	**Other immunotherapy group (*N* = 213)**	**DMT treatment group (*N* = 111)**	**Odds ratio (95% confidence interval)**	***P*-value**
Gender (female%)	62.98% (131/208)	77.27% (85/110)	2.000 (1.179–3.390)	*P* = 0.01
Age, median (IQR), years	40.50 (19.05) (*N* = 207)	37.45 (16.12) (*N* = 103)	0.984 (0.966–1.002)	*P* > 0.05
Married (%)	77.29% (160/207)	68.18% (75/110)	1.589 (0.948–2.663)	*P* > 0.05
Procreation (%)	73.27% (148/202)	63.64% (70/110)	1.468 (0.897–2.403)	*P* > 0.05
Education level (%)				*P* < 0.0001
Primary education	8.96% (18/201)	5.45% (6/110)	1	
Junior high school	26.37% (53/201)	7.27% (8/110)	0.453 (0.138–1.482)	
High school	27.36% (55/201)	23.64% (26/110)	1.418 (0.504–3.992)	
Bachelor's degree	37.31% (75/201)	63.64% (70/110)	2.800 (1.051–7.458)	

*DMT, disease-modifying therapy; IQR, interquartile range*.

In terms of disease characteristics, the course of the disease, diagnosis, EDSS scores, and the number of symptoms were not statistically correlated with the treatment choice. It was clear from the analysis that among the 14 symptoms, only limb weakness, pain, and fatigue influenced the patient's choice of treatment with DMTs. Patients with limb weakness and pain were less likely to accept DMTs, whereas patients with fatigue were 2.083 (95% CI: 1.299–3.344) times more likely to accept DMTs than patients without this symptom (Table 5).

**Table 5:** Disease characteristics of the DMT treatment group and other immunotherapy group.

**Variables**	**Other immunotherapy group (*N* = 213)**	**DMT treatment group (*N* = 111)**	**Odds ratio (95% confidence interval)**	***P*-value**
Course of diseases, median (IQR), years	5.90 (7.22) (*N* = 172)	8.32 (5.34) (*N* = 87)	1.030 (0.988–1.073)	*P* > 0.05
Diagnosis (%)	(*N* = 141)	(*N* = 55)	–	*P* > 0.05
EDSS, median (IQR)	2.5 (4) (*N* = 119)	2 (2.875) (*N* = 42)	1.003 (0.995–1.010)	*P* > 0.05
Symptoms (%)				
Hypoxia	47.42% (101/213)	42.34% (47/111)	0.814 (0.513–1.294)	*P* > 0.05
Amblyopia	30.52% (65/213)	27.03% (30/111)	0.843 (0.506–1.404)	*P* > 0.05
Limb weakness	69.01% (147/213)	55.86% (62/111)	0.568 (0.354–0.912)	*P* = 0.019
Dizziness	34.74% (74/213)	29.73% (33/111)	0.795 (0.484–1.304)	*P* > 0.05
Emotional disorder	38.97% (83/213)	34.23% (38/111)	0.815 (0.505–1.316)	*P* > 0.05
Unstable walking	51.17% (109/213)	53.15% (59/111)	1.082 (0.684–1.715)	*P* > 0.05
Muscle spasms	31.46% (67/213)	28.83% (32/111)	0.883 (0.534–1.458)	*P* > 0.05
Pain	25.35% (54/213)	13.51% (15/111)	0.460 (0.246–0.860)	*P* = 0.015
Sexual dysfunction	9.86% (21/213)	10.81% (12/111)	1.109 (0.524–2.347)	*P* > 0.05
Dysphonia	20.66% (44/213)	16.22% (18/111)	0.743 (0.406–1.361)	*P* > 0.05
Fecal and urine	32.39% (69/213)	32.43% (36/111)	1.002 (0.613–1.637)	*P* > 0.05
incontinence				
Paresthesia	59.15% (126/213)	62.16% (69/111)	1.134 (0.708–1.818)	*P* > 0.05
Memory impairment	29.58% (63/213)	26.13% (29/111)	0.842 (0.503–1.410)	*P* > 0.05
Fatigue	46.01% (98/213)	63.96% (71/111)	2.083 (1.299–3.344)	*P* = 0.002
Number of symptoms			1.030 (0.958–1.109)	*P* > 0.05
<2	8.45% (18/213)	14.41% (16/111)		
≥2 to <4	26.76% (57/213)	25.23% (28/111)		
≥4 to <8	42.25% (90/213)	36.04% (40/111)		
≥8	22.54% (48/213)	24.32% (27/111)		

*DMT, disease-modifying therapy; IQR, interquartile range; EDSS, Expanded Disability Status Scale*.

**Figure 3:**
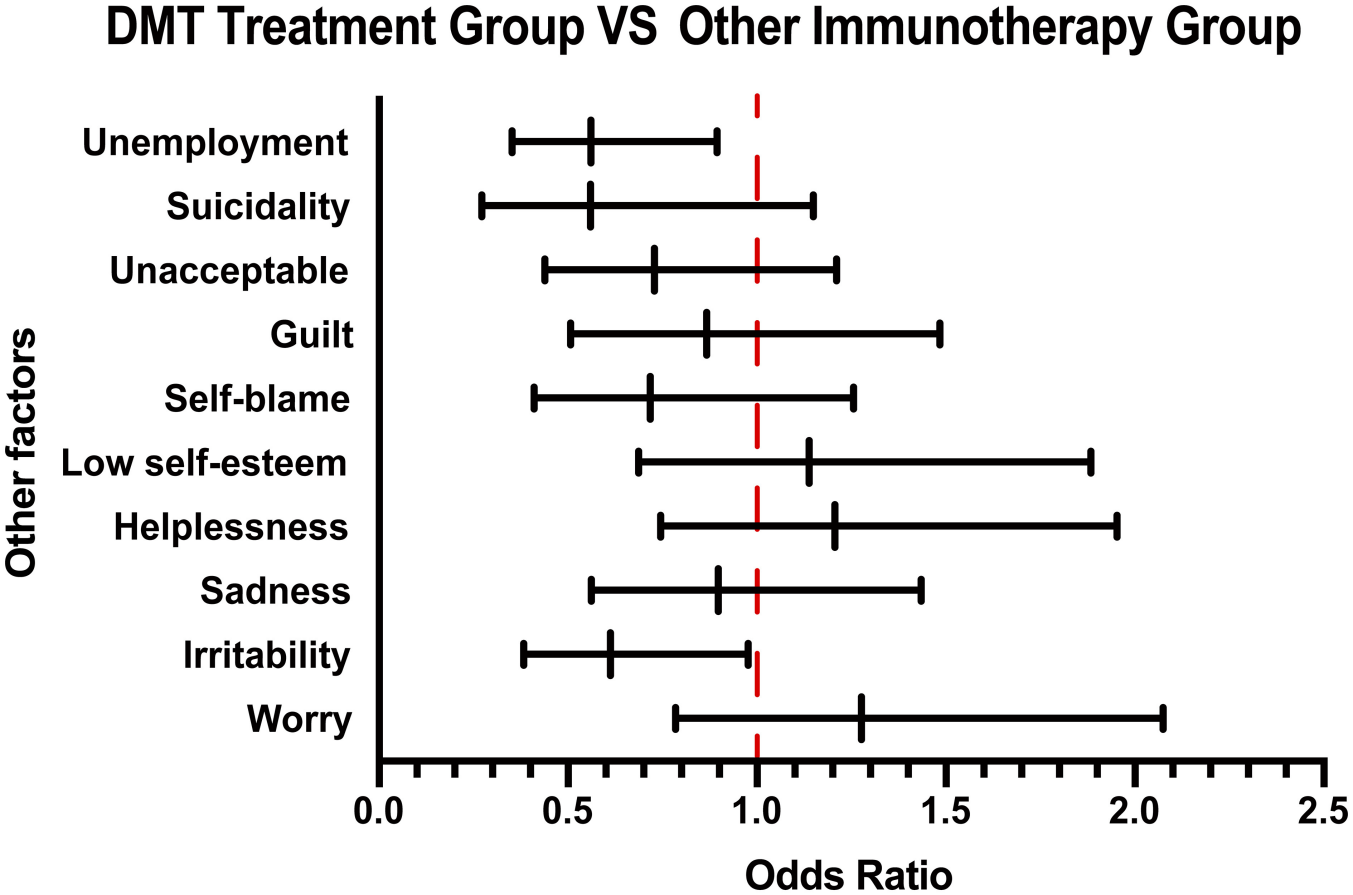
Other emotional and employment factors differing between the DMT-treated group and other immunotherapy group. The forest plot shows odds ratio differences in other factors between the DMT treatment group and other immunotherapy groups. DMT, disease-modifying therapy.

On the basis of the 10-question analysis, we found that patients in the DMT treatment group generally scored higher than those in the group that received other immunotherapy treatment (Supplementary Table 2). As for the psychological and economic burden of the disease, by analyzing the patients' emotional response, we found that there was a higher proportion of irritability in the group receiving other immunotherapy treatment than the DMT treatment group. Moreover, an analysis of their employment status showed that the unemployment rate of patients receiving other immunotherapy treatment was higher than that of patients receiving DMT treatment, suggesting that the patients' income affects the treatment choice (Figure 3).

### DMT Treatment Group vs. Untreated Group

To analyze the characteristics of patients using DMTs, we further compared the DMT-treated patients with untreated patients. In terms of basic characteristics, the proportion of female patients was higher in the DMT treatment group, and all the basic characteristics such as age and educational level were significantly associated with the patients treated with DMTs. In addition, unmarried patients were 1.935 (95% CI: 1.249–2.998) times more likely to accept DMTs than married patients, and patients without children were 1.677 (95% CI: 1.101–2.553) times more likely to accept DMTs than those with children, suggesting the impact of family burden in the acceptance of treatment (Table 6).

**Table 6:** Patient characteristics of the DMT treatment group and untreated group.

**Variables**	**Untreated group (*N* = 888)**	**DMT treatment group (*N* = 111)**	**Odds ratio (95% confidence interval)**	***P*-value**
Gender (female%)	66.87% (575/860)	77.27% (85/110)	1.686 (1.055–2.688)	*P* = 0.029
Age, median (IQR), years	41.42 (18.58) (*N* = 798)	37.45 (16.12) (*N* = 103)	0.977 (0.961–0.993)	*P* = 0.005
Married (%)	80.57% (647/803)	68.18% (75/110)	1.935 (1.249–2.998)	*P* = 0.003
Procreation (%)	74.58% (584/783)	63.64% (70/110)	1.677 (1.101–2.553)	*P* = 0.016
Education level (%)				*P* < 0.0001
Primary education	7.94% (61/768)	5.45% (6/110)	1	
Junior high school	29.43% (226/768)	7.27% (8/110)	0.360 (0.120–1.076)	
High school	29.69% (228/768)	23.64% (26/110)	1.159 (0.457–2.943)	
Bachelor's degree	32.94% (253/768)	63.64% (70/110)	2.813 (1.167–6.777)	

*DMT, disease-modifying therapy; IQR, interquartile range*.

As shown in Table 7, diagnosis and EDSS scores were not statistically correlated with treatment choice. However, the course of disease, type of symptoms, and the number of symptoms influence the patient's choice of treatment with DMTs. Specifically, patients having longer course of disease and more symptoms were likely to use DMTs, indicating that patients more affected by the disease are more willing to be treated.

**Table 7:** Disease characteristics of the DMT treatment group and untreated group.

**Variables**	**Untreated group (*N* = 888)**	**DMT treatment group (*N* = 111)**	**Odds ratio (95% confidence interval)**	***P*-value**
Course of diseases, median (IQR), years	6.15 (6.59) (*N* = 569)	8.32 (5.34) (*N* = 87)	1.043 (1.006–1.081)	*P* = 0.022
Diagnosis (%)	(*N* = 644)	(*N* = 55)	–	*P* > 0.05
EDSS, median (IQR)	2 (2.5) (*N* = 566)	2 (2.875) (*N* = 42)	−0.001 (−0.011–0.008)	*P* > 0.05
Symptoms (%)				
Hypoxia	25.90% (230/888)	42.34% (47/111)	2.101 (1.401–3.155)	*P* < 0.0001
Amblyopia	17.55% (156/888)	27.03% (30/111)	1.751 (1.114–2.755)	*P* = 0.015
Limb weakness	40.09% (356/888)	55.86% (62/111)	1.890 (1.271–2.817)	*P* = 0.002
Dizziness	23.76% (211/888)	29.73% (33/111)	1.357 (0.878–2.096)	*P* > 0.05
Emotional disorder	17.57% (156/888)	34.23% (38/111)	2.445 (1.592–3.745)	*P* < 0.0001
Unstable walking	27.70% (246/888)	53.15% (59/111)	2.959 (1.984–4.425)	*P* < 0.0001
Muscle spasms	11.15% (102/888)	28.83% (32/111)	3.226 (2.037–5.128)	*P* < 0.0001
Pain	16.10% (143/888)	13.51% (15/111)	0.814 (0.459–1.443)	*P* > 0.05
Sexual dysfunction	4.84% (43/888)	10.81% (12/111)	2.381 (1.215–4.673)	*P* = 0.011
Dysphonia	9.80% (87/888)	16.22% (18/111)	1.783 (1.027–3.096)	*P* = 0.04
Fecal and urine incontinence	16.67% (148/888)	32.43% (36/111)	2.398 (1.553–3.704)	*P* < 0.0001
Paresthesia	42.91% (381/888)	62.16% (69/111)	2.188 (1.458–3.279)	*P* < 0.0001
Memory impairment	14.64% (130/888)	26.13% (29/111)	2.062 (1.299–3.279)	*P* = 0.002
Fatigue	30.07% (267/888)	63.96% (71/111)	4.132 (2.732–6.25)	*P* < 0.0001
Number of symptoms			1.211 (1.141–1.285)	*P* < 0.0001
<2	36.49% (324/888)	14.41% (16/111)		
≥2 to <4	31.53% (280/888)	25.23% (28/111)		
≥4 to <8	23.09% (205/888)	36.04% (40/111)		
≥8	8.89% (79/888)	24.32% (27/111)		

*DMT, disease-modifying therapy; IQR, interquartile range; EDSS, Expanded Disability Status Scale*.

**Figure 4:**
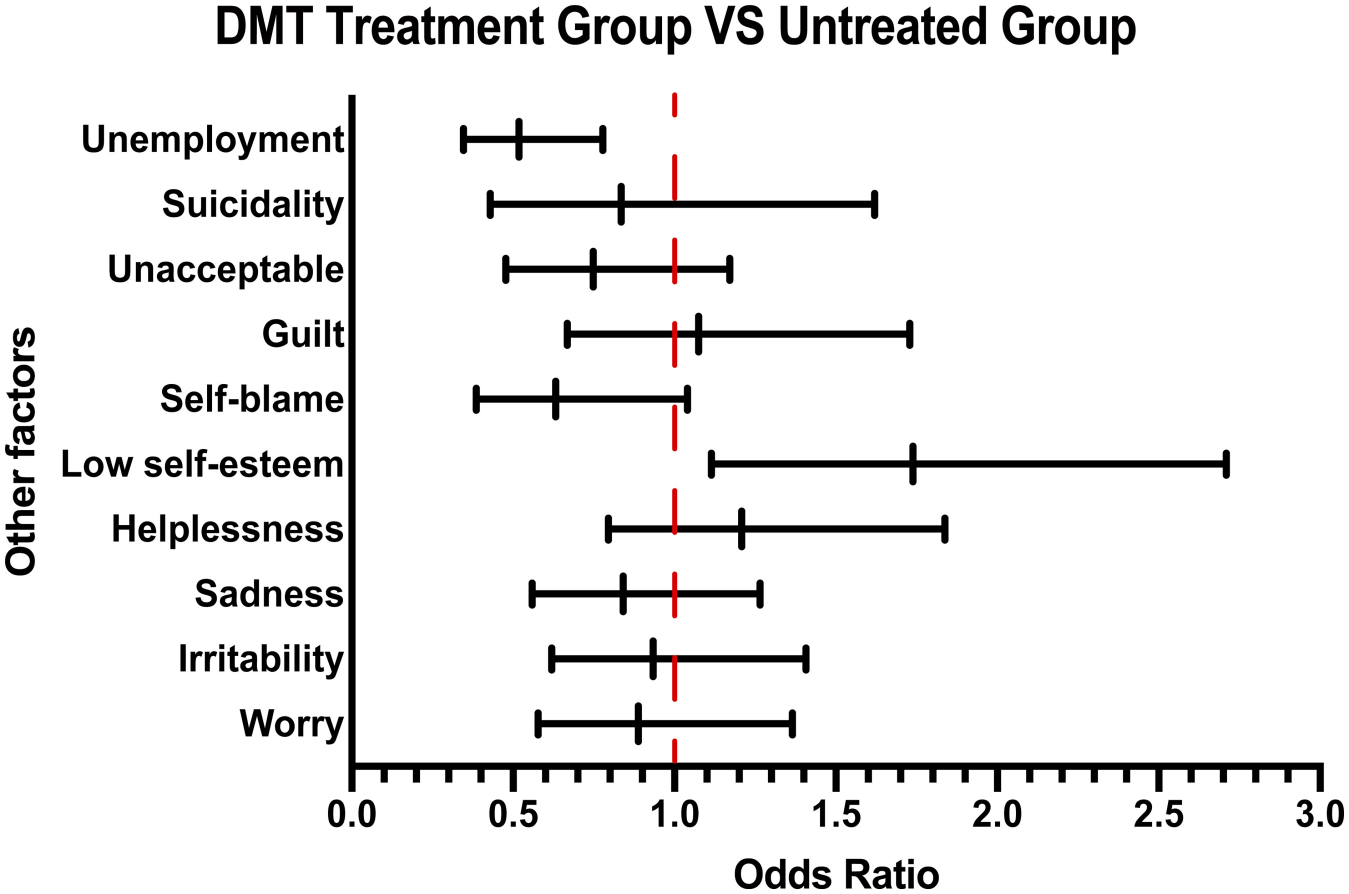
Other emotional and employment factors differing between the DMT-treated group and untreated group. The forest plot shows odds ratio differences in other factors between the DMT treatment group and untreated group.

Consistent with previous results, patients in the DMT treatment group generally scored higher during the 10-question survey than those in the untreated group, suggesting that patients with more background knowledge about disease would prefer treatment (Supplementary Table 3). In terms of the psychological and economic burden of the disease, by analyzing their emotional response, a higher proportion of low self-esteem was found in the DMT treatment group compared with the untreated group. Moreover, investigation of their employment status showed that the unemployment rate of untreated patients was higher than that of the patients receiving DMT treatment, suggesting that patients' income affects their drug acceptance (Figure 4).

## Discussion

In our study, we found that elderly, married, and unemployed patients were less likely to receive the treatment. This can be attributed to the huge economic cost the treatment incurs to the patients and their families (16–18). The total cost associated with MS in Spain was estimated as €1.395 million per year, and €30,050 was the mean annual cost per patient (19). Similar results were seen in the Finnish population where the mean annual cost per patient was estimated to be €4,699,427 (20). The largest cost component for individuals with MS are prescription drugs, specifically the DMTs, which are responsible for acquisition cost of more than $70,000 a year in the USA (21).

In China, according to the National Bureau of Statistics, the per capita disposable income of residents was only €3,325 in 2017, whereas as the *Multiple Sclerosis Patient Survival Report 2018* illustrated, roughly 61% of the patients with MS incurred more than €1,277 in medical costs per relapse, suggesting the cost of each recurrence is one-third of China's per capita income in 2017, and moreover, ~25% of the patients lose their ability to work after illness. Fortunately, MS was included in the first list of rare diseases issued by the National Health Council of China in May 2018, giving MS the unprecedented attention (22).

In addition to financial burden, the second important reason for patients' refusal to treatment was when their symptoms are mild. As shown in our study, patients suffering from a long course of disease and various symptoms were more likely to accept the treatment. It is undeniable that many patients with MS ignore the progress of the disease because inflammation does not always result in a relapse or visible symptoms, but they may still experience deterioration caused by MS that can only be seen on a brain scan (23, 24). With the passage of time, the loss of function is so gradual that it goes unnoticed by the patient or physician. Moreover, these patients have low EDSS scores and normal motor function, which do not fully reflect subjective symptoms (25). In our survey, we found that patients with higher awareness of the disease or with higher education were more likely to accept the treatment than those with lower qualification. Previously conducted studies showed that patients with higher education were more likely to actively cooperate with doctors to receive drug treatment (26, 27), and patient education and awareness play a very important role in the management of diseases (28) and treatment adherence (29). This suggests that neurologists should carefully educate patients with MS about the disease. Only a full understanding of the characteristics and prognosis of the disease and the importance of DMTs in the treatment of MS can help the patients make a reasonable choice, especially for patients with poor education level.

We found that patients with frequent relapses were more willing to be treated, and many patients did not receive proper treatment in their CIS stage. In Spain, the median time from onset of first symptoms to the first visit to a physician was 19.2 months, which represented the greatest delay (30), and in the Portuguese population, the median time between the first clinical manifestation and MS diagnosis was merely 9 months (31). However, on the basis of data from the *Multiple Sclerosis Patient Survival Report 2018*, the situation of delayed diagnosis and treatment is worrisome in China, with merely 53% of the patients being diagnosed and treated immediately. Moreover, 65% of the patients took ~1–5 years to seek help from neurologists, and about 8% of the patients did not go to hospitals until after 6 years of illness. Similarly, 65% of the patients took roughly 1–5 years to be diagnosed with MS, and this period was more than 6 years for 12% of the patients. There are many factors that are responsible for this result, of which the lack of understanding of the disease by neurologists themselves is a factor that cannot be ignored. CIS is the first episode of neurologic symptoms caused by demyelination and inflammation in the central nervous system, and the conversion rate from CIS to MS was 79% (32). Several studies have demonstrated that DMTs work effectively to reduce the accumulation of neural cell damage when the patient starts treatment as soon as possible after diagnosis and especially before the appearance of any signs of disability (33, 34). More importantly, DMTs appear to attenuate brain atrophy over time in the patients with CIS (35) and delay further episodes that would lead to a definite diagnosis of MS (36). Therefore, neurologists need to enhance their understanding of the disease and update their learning on recent advances in the disease.

In our study, only 10 patients were treated with rituximab. However, because rituximab has not been approved by the European Medicine Agency, the US FDA, or the China National Medical Products Administration as a disease-modifying drug for the treatment of MS (37, 38), and the *Chinese Expert Consensus on Diagnosis and Treatment of Multiple Sclerosis (2018 Edition)* does not recommend rituximab as a disease-modifying drug for MS (39), rituximab is off-label for the treatment of patients with MS and was included in the other immunotherapy treatment group. On the other hand, as a humanized monoclonal antibody, the price of rituximab far exceeds the acceptance of most patients, and national health insurance does not cover this imported novel drug. Therefore, the accessibility of rituximab in Chinese patients with MS is poor.

There are several shortcomings in the present study. First, in view of the immature market of disease-modifying drugs in China, the number of patients with MS using DMTs is less; second, more indicators to assess the economic impact should be included, and with regard to the patient's privacy, using interviews to investigate their financial burden would be more effective than questionnaires; third, more assessment scales such as quality of life (QoL), European Quality of Life Five Dimension (EuroQol-5D), and Hamburg Quality of Life Questionnaire Multiple Sclerosis (HAQUAMS) should be included to ensure the accuracy of the study to assess patient's quality of life. However, this investigation of the characteristics of Chinese patients with MS is meaningful for clinical work. Our study found that patient acceptance and choice of therapy are affected by many factors such as age, sex, marriage, education level, disease subtypes, symptoms, course of disease, mood, employment, and understanding of the disease, among which the most important factors are education level and understanding of the disease, suggesting that patient education should be included as part of the treatment process.

As of August 2020, 2,854 large comprehensive hospitals across the country have set up neurology specialties (40), and in November 2020, the Neuroimmunology Branch of Chinese Society of Immunology selected 20 hospitals as Chinese Multiple Sclerosis Diagnosis and Treatment Center. In addition, to accelerate the marketing speed of drugs for rare diseases, China has established a fast track for qualified drugs urgently needed for rare diseases that have been marketed overseas. On July 13, 2017, the Ministry of Human Resources and Social Security of the People's Republic of China approved Betaferon to enter the reimbursement scope of national medical insurance and gave 28.9% price reduction to make it more accessible to patients (41). Moreover, it is encouraging that teriflunomide was allowed to enter the Chinese market in 2018, and the indications for CIS in China were approved in September 2020. In addition, fingolimod and siponimod have recently entered the Chinese market, which will provide more options for Chinese patients with MS. With the promulgation of policies, the update of doctors' understanding of rare diseases, and the marketing of new drugs, the main factors that affect the patients' acceptance of medical treatment will be changed, and more attention may be paid to the disease-modifying drugs' properties, such as the efficacy and side effects of the drugs. Therefore, it is necessary to reconduct a large-scale survey on the quality of life of Chinese patients with MS in the near future with a larger patient population and inclusion of the patients' income to obtain a more comprehensive conclusion.

## Data Availability Statement

The original contributions presented in the study are included in the article/Supplementary Material, further inquiries can be directed to the corresponding author/s.

## Ethics Statement

The studies involving human participants were reviewed and approved by the Medical Research Ethics Committee of Xiangya Hospital and conducted in accordance to the Declaration of Helsinki. The patients/participants provided their written informed consent to participate in this study.

## Author Contributions

HY and QZ conceived the presented idea and verified the analytical methods. RZ performed the computations and wrote the manuscript. YX, GT, HL, LWa, HZ, MeZ, JF, TJ, XZ, HY, JW, XuZ, FG, CY, BB, CL, MiZ, HD, AL, WL, LWu, MW, YT, HW, YL, ZW, and WZ ensured the quality of this study and provided source of data. All authors contributed to the article and approved the submitted version.

## Conflict of Interest

The authors declare that the research was conducted in the absence of any commercial or financial relationships that could be construed as a potential conflict of interest.
