# Supplementary material for: Status of Immunotherapy Acceptance in Chinese Patients With Multiple Sclerosis: Analysis of Multiple Sclerosis Patient Survival Report 2018
Source: Front Neurol. 2021 Apr 8;12:651511. doi: 10.3389/fneur.2021.651511 (PMC8060470; doi:10.3389/fneur.2021.651511)
Supplement: Supplementary file 1 [file Data_Sheet_1.docx]

# Supplementary Tables

**Supplementary Table 1. The Number of Patients Who Answered Questions Incorrectly and Correctly in Both the Treated and Untreated Groups.** Q1: Multiple sclerosis is a chronic disease affecting the central nervous system that is not yet completely cured and can only try to delay disease progression; Q2: The symptoms of multiple sclerosis vary widely, and new symptoms may appear each time they recur, but some symptoms persist for a long time; Q3: Multiple sclerosis will have irreversible brain or spinal cord damage each time it recurs; Q4: The absence of relapse in multiple sclerosis does not represent the absence of disease progression, and damage to the brain or spinal cord occurs even in the absence of relapse; Q5: Patients with multiple sclerosis have a higher incidence of cognitive deficits and brain atrophy than the general population; Q6: Life expectancy of patients with multiple sclerosis will be shortened; Q7: Multiple sclerosis can lead to disability, and many patients with multiple sclerosis will eventually need to walk on assistive tools such as crutches and wheelchairs, or stay in bed all day long; Q8: The ultimate goal of multiple sclerosis treatment is to reduce relapses and delay disability progression; Q9: Low-dose hormones (referring to glucocorticoids) are of little significance in reducing relapse and delaying disability progression; Q10: Long-term use of azathioprine will increase the risk of tumors.

| **Variables** | **Untreated Group**  **(N=888)** | **Treated Group**  **(N=324)** | **Odds Ratio**  **(95% Confidence Interval)** | ***P* value** |
| --- | --- | --- | --- | --- |
| **Multiple Sclerosis-Related Problems (%)** |  |  |  |  |
| Q1 | 88.76% (616/694) | 92.88% (300/323) | 1.652(1.017-2.683) | *P*=0.043 |
| Q2 | 83.53% (578/692) | 89.44% (288/322) | 1.669(1.111-2.513) | *P*=0.014 |
| Q3 | 77.08% (528/685) | 80.69% (259/321) | 1.242(0.894-1.727) | *P*>0.05 |
| Q4 | 64.90% (442/681) | 67.61% (215/318) | 1.129(0.851-1.497) | *P*>0.05 |
| Q5 | 65.76% (436/663) | 72.19% (231/320) | 1.351(1.009-1.812) | *P*=0.044 |
| Q6 | 61.59% (404/656) | 62.07% (198/319) | 1.020(0.775-1.344) | *P*>0.05 |
| Q7 | 75.15% (514/684) | 83.07% (265/319) | 1.623(1.155-2.283) | *P*=0.005 |
| Q8 | 83.04% (558/672) | 93.46% (300/321) | 2.915(1.795-4.739) | *P*<0.0001 |
| Q9 | 60.53% (408/674) | 64.35% (204/317) | 1.176(0.892-1.553) | *P*>0.05 |
| Q10 | 52.06% (341/655) | 67.43% (205/304) | 1.908(1.435-2.532) | *P*<0.0001 |
| **Number of correct responses (%)** |  |  | 0.971(0.902-1.044) | *P*<0.0001 |
| <6 | 23.74% (146/615) | 14.76% (44/298) |  |  |
| ≥6 to <8 | 23.25% (143/615) | 22.15% (66/298) |  |  |
| >8 | 53.01% (326/615) | 63.09% (188/298) |  |  |

**Supplementary Table 2. The Number of Patients Who Answered Questions Incorrectly and Correctly in Both the DMT Treatment Group and Other Immunotherapy Group**

| **Variables** | **Other Immunotherapy Group**  **(N=213)** | **DMT Treatment Group**  **(N=111)** | **Odds Ratio**  **(95% Confidence Interval)** | ***P* value** |
| --- | --- | --- | --- | --- |
| **Multiple Sclerosis**  **Related Problems (%)** |  |  |  |  |
| Q1 | 90.61% (193/213) | 97.27% (107/110) | 3.690(1.074-12.658) | *P*=0.038 |
| Q2 | 87.32% (186/213) | 93.58% (102/109) | 2.114(0.890-5.025) | *P*>0.05 |
| Q3 | 76.30% (161/211) | 89.09% (98/110) | 2.538(1.287-5.00) | *P*=0.007 |
| Q4 | 62.09% (134/208) | 77.06% (81/110) | 1.543(0.926-2.571) | *P*>0.05 |
| Q5 | 69.67% (147/211) | 77.06% (84/109) | 1.462(0.858-2.494) | *P*>0.05 |
| Q6 | 64.29% (135/210) | 57.80% (63/109) | 0.761(0.474-1.221) | *P*>0.05 |
| Q7 | 80.95% (170/210) | 87.16% (95/109) | 1.597(0.826-3.086) | *P*>0.05 |
| Q8 | 91.00% (192/211) | 98.18% (108/110) | 5.348(1.221-23.256) | *P*=0.026 |
| Q9 | 60.29% (126/209) | 72.22% (78/108) | 1.712(1.034-2.833) | *P*=0.036 |
| Q10 | 64.36% (130/202) | 73.53% (75/102) |  |  |
| **Number of correct responses (%)** |  |  | 1.125(1.014-1.248) | *P*=0.026 |
| <6 | 19.19% (38/198) | 6.00% (6/100) |  |  |
| ≥6 to <8 | 23.23% (46/198) | 20.00% (20/100) |  |  |
| >8 | 57.58% (114/198) | 74.00% (74/100) |  |  |

**Supplementary Table 3. The Number of Patients Who Answered Questions Incorrectly and Correctly in Both the DMT Treatment Group and Untreated Group**

| **Variables** | **Untreated Group**  **(N=888)** | **DMT Treatment Group**  **(N=111)** | **Odds Ratio**  **(95% Confidence Interval)** | ***P* value** |
| --- | --- | --- | --- | --- |
| **Multiple Sclerosis-Related Problems (%)** |  |  |  |  |
| Q1 | 88.76% (616/694) | 97.27% (107/110) | 4.525(1.401-14.493) | *P*=0.012 |
| Q2 | 83.53% (578/692) | 93.58% (102/109) | 2.874(1.302-6.329) | *P*=0.009 |
| Q3 | 77.08% (528/685) | 89.09% (98/110) | 2.427(1.299-4.545) | *P*=0.005 |
| Q4 | 64.90% (442/681) | 77.06% (81/110) | 1.511(0.961-2.375) | *P*>0.05 |
| Q5 | 65.76% (436/663) | 77.06% (84/109) | 1.748(1.088-2.809) | *P*=0.021 |
| Q6 | 61.59% (404/656) | 57.80% (63/109) | 0.854(0.566-1.289） | *P*>0.05 |
| Q7 | 75.15% (514/684) | 87.16% (95/109) | 2.242(1.247-4.032) | *P*=0.007 |
| Q8 | 83.04% (558/672) | 98.18% (108/110) | 10.989(2.688-4.545) | *P*=0.001 |
| Q9 | 60.53% (408/674) | 72.22% (78/108) | 1.695(1.082-2.653) | *P*=0.021 |
| Q10 | 52.06% (341/655) | 73.53% (75/102) |  |  |
| **Number of correct responses (%)** |  |  | 1.316(1.210-1.432) | *P*<0.0001 |
| <6 | 23.74% (146/615) | 6.00% (6/100) |  |  |
| ≥6 to <8 | 23.25% (143/615) | 20.00% (20/100) |  |  |
| >8 | 53.01% (326/615) | 74.00% (74/100) |  |  |
